# Supplementary material for: Peripheral nerve resident macrophages share tissue-specific programming and features of activated microglia
Source: Nat Commun. 2020 May 21;11:2552. doi: 10.1038/s41467-020-16355-w (PMC7242366; doi:10.1038/s41467-020-16355-w)
Supplement: Supplementary file 1 — Supplementary Information [file 41467_2020_16355_MOESM1_ESM.pdf]

**Supplementary Figure 1. Analysis of resident macrophage chimerism in CD45.1 wild type and CD45.2 Lyz2-Cre tdTomato parabionts.**

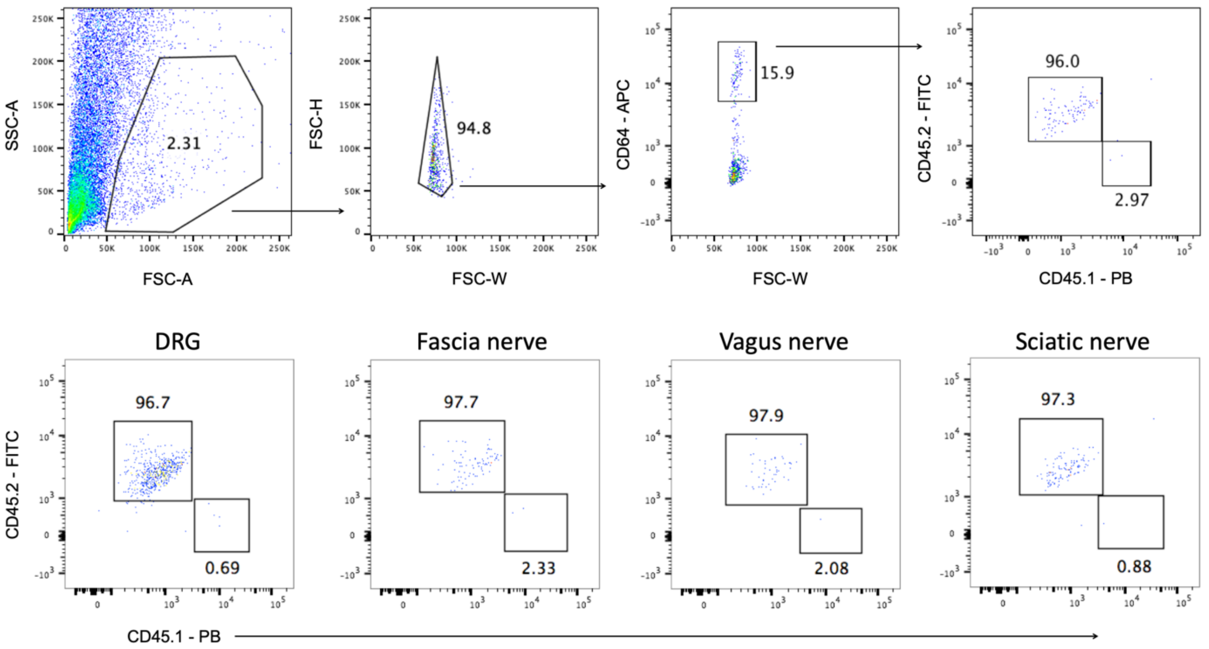


**Supplementary Figure 1. Analysis of resident macrophage chimerism in CD45.1 wild type and CD45.2 Lyz2-Cre tdTomato parabionts.** Gating scheme (top) and representative flow plots of CD45.1 and CD45.2 expression in nerve macrophages from CD45.2 parabiont (bottom).

**Supplementary Figure 2. Flow cytometric analysis of blood and neural resident macrophages in pulse chase experiment (related to Figure 1j-l)**

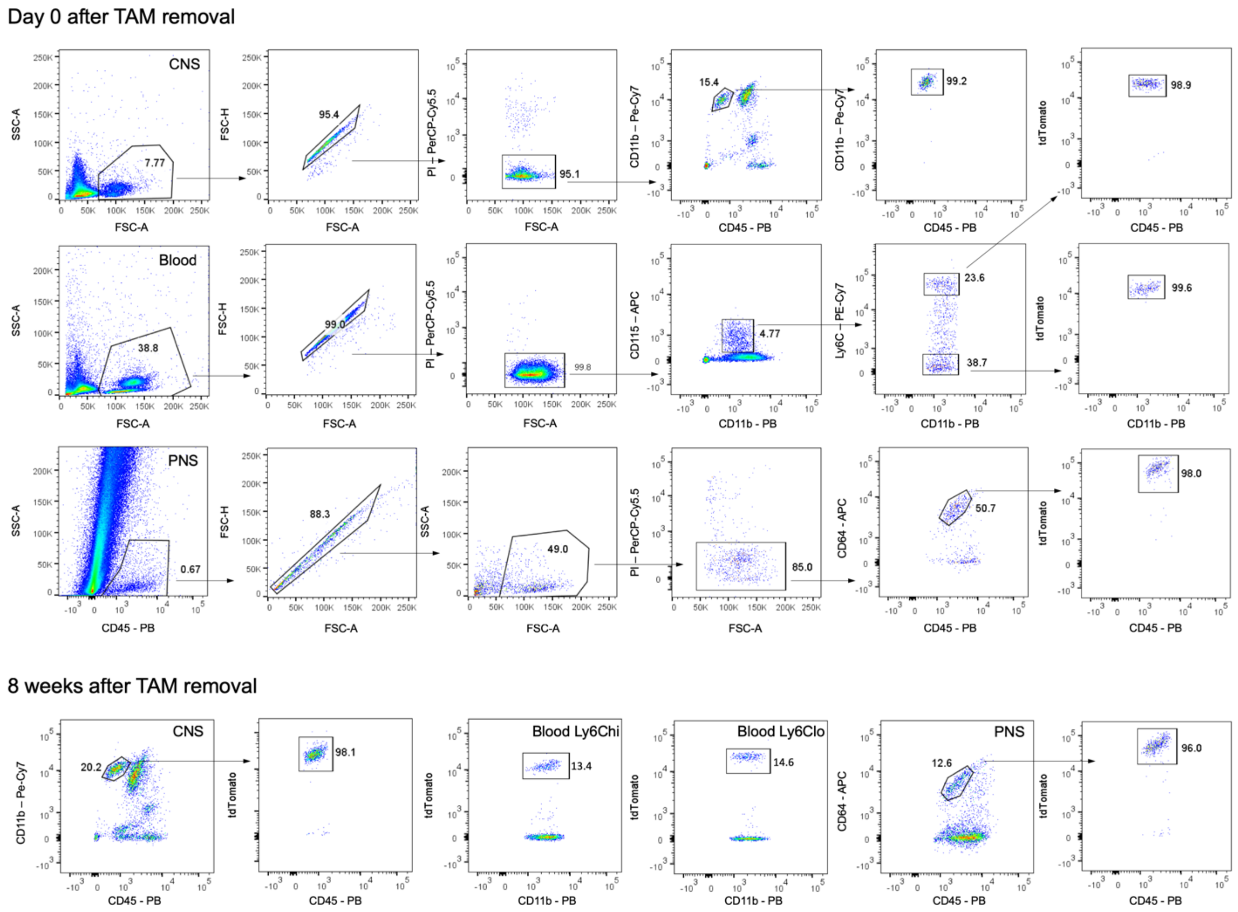


**Supplementary Figure 2. Flow cytometric analysis of blood and neural resident macrophages in pulse chase experiment (related to Figure 1j-l)**. Gating strategy and representative flow plots of CNS microglia, blood monocytes, and PNS macrophages at day 0 and 8 weeks after tamoxifen removal.

**Supplementary Figure 3. Gating strategy for identifying PNS macrophages (related to Figure 1d) and for double-sorted populations used in bulk-RNA sequencing**

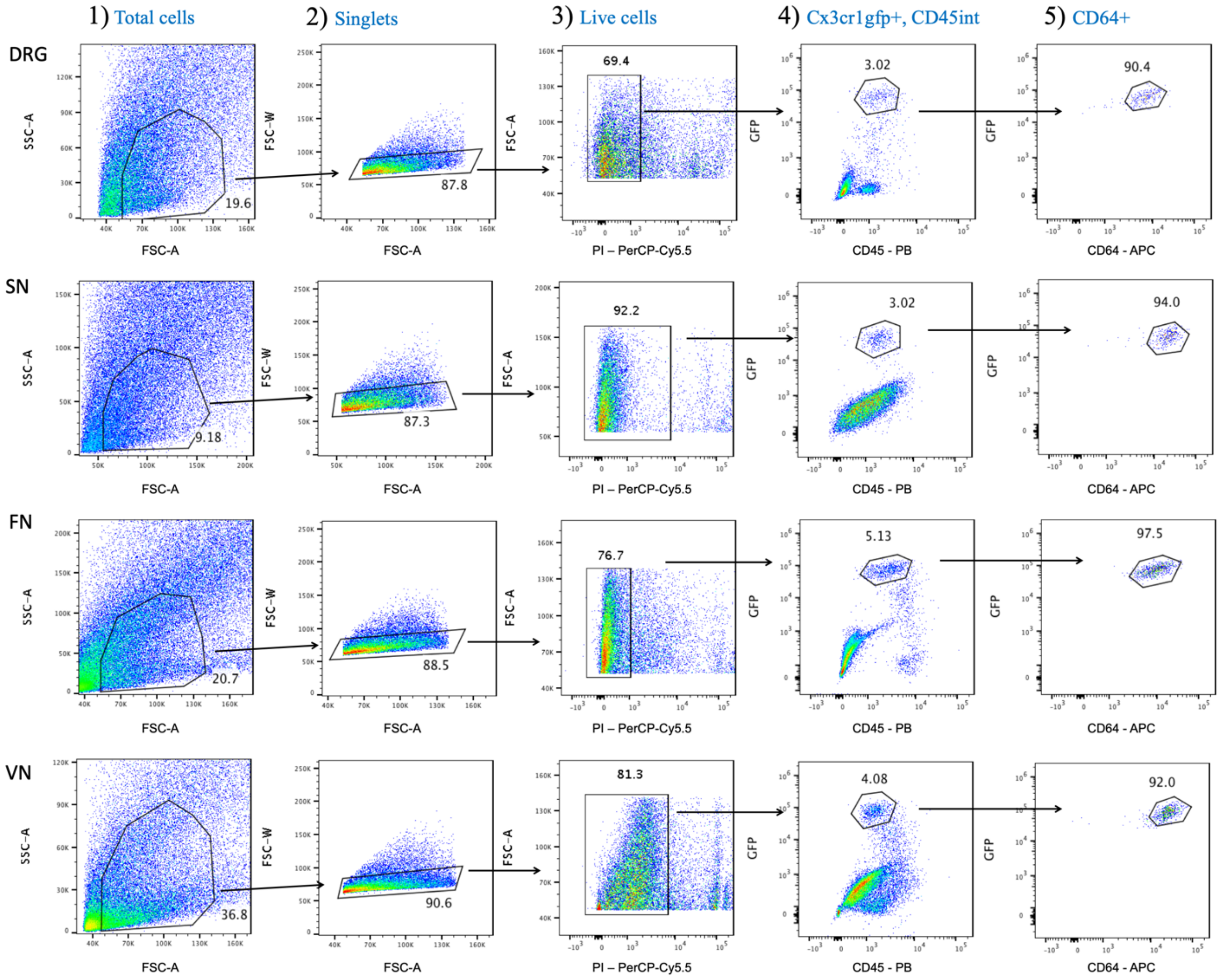


**Supplementary Figure 3. Gating strategy for identifying PNS macrophages (related to Figure 1d) and for double-sorted populations used in bulk-RNA sequencing**. Representative gating schematic for flow cytometric identification and sorting of CX3CR1-GFP+, CD45 intermediate, CD64+ PNS macrophages in DRG, SN, FN, and VN .

**Supplementary Figure 4. Uniquely downregulated genes in neural resident macrophages**

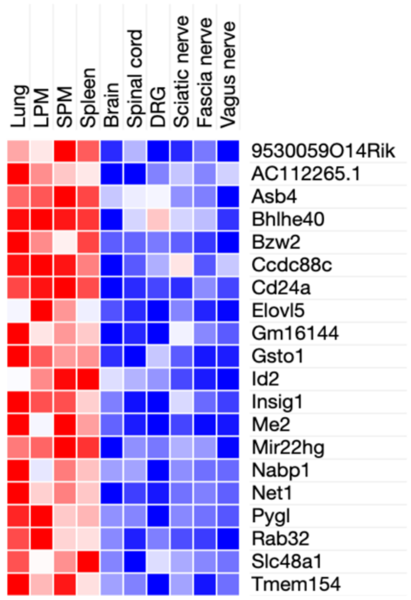


**Supplementary Figure 4. Uniquely downregulated genes in neural resident macrophages**. Heat map of mRNA transcripts fourfold or more downregulated in CNS microglia and PNS macrophages compared to conventional tissue-resident macrophages. Each box represents average of three replicates.

**Supplementary Figure 5. Sall1 expression in PNS macrophages**

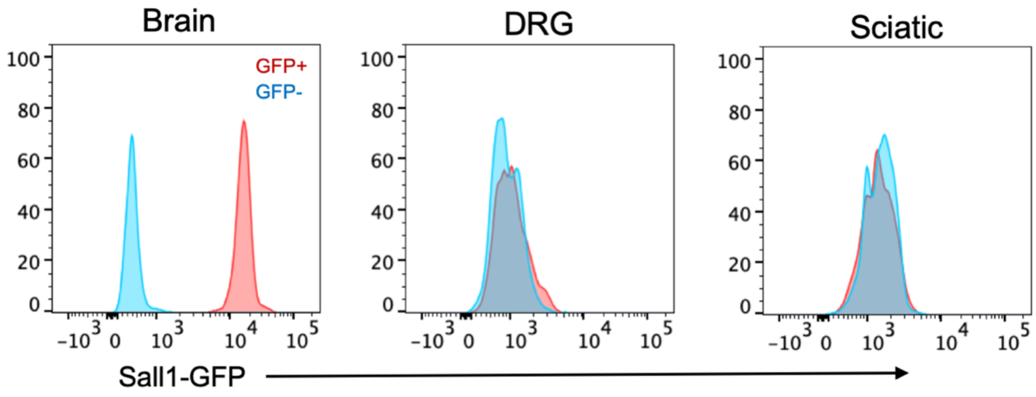


**Supplementary Figure 5. Sall1 expression in PNS macrophages**. Flow cytometry analysis of GFP expression in brain microglia, DRG macrophages, and sciatic nerve macrophages isolated from Sall1-GFP/+ mice (red) and wild type controls (blue).

**Supplementary Figure 6. Identification of unique signatures in CNS microglia**

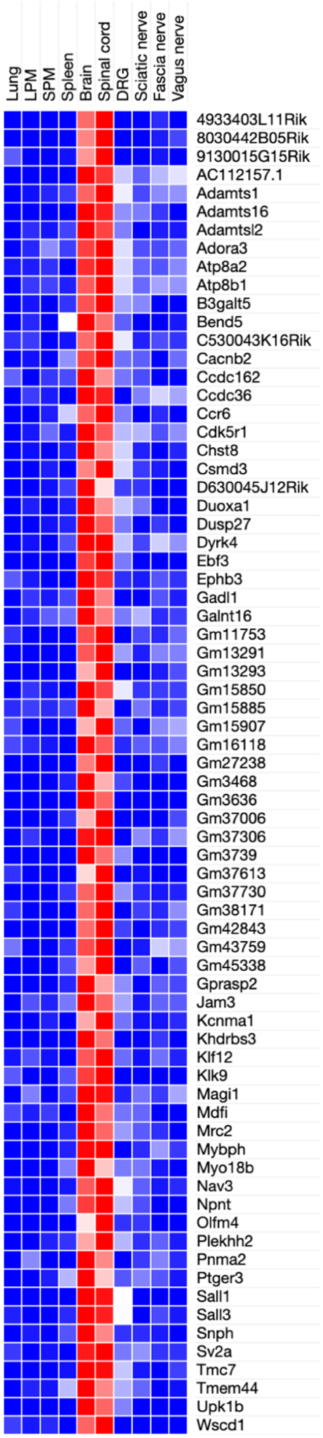


**Supplementary Figure 6. Identification of unique signatures in CNS microglia**. Heat map of transcripts selectively enriched fourfold or more in brain and spinal cord microglia compared to PNS and conventional macrophages. Each box represents average of three replicates.

**Supplementary Figure 7. Downregulated genes in PNS macrophages**

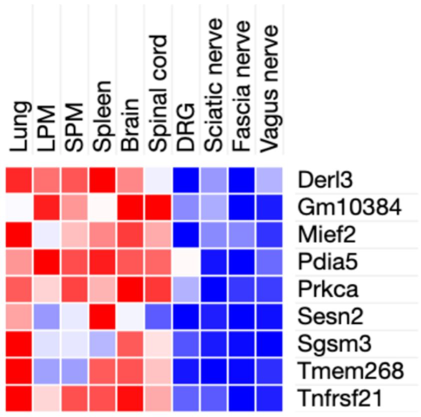


**Supplementary Figure 7. Downregulated genes in PNS macrophages**. Heat map of genes that are downregulated fourfold or more in PNS macrophages compared to all other populations combined. Each box represents average of three replicates.

## Supplementary Figure 8. Unique gene expression patterns in individual PNS macrophage populations

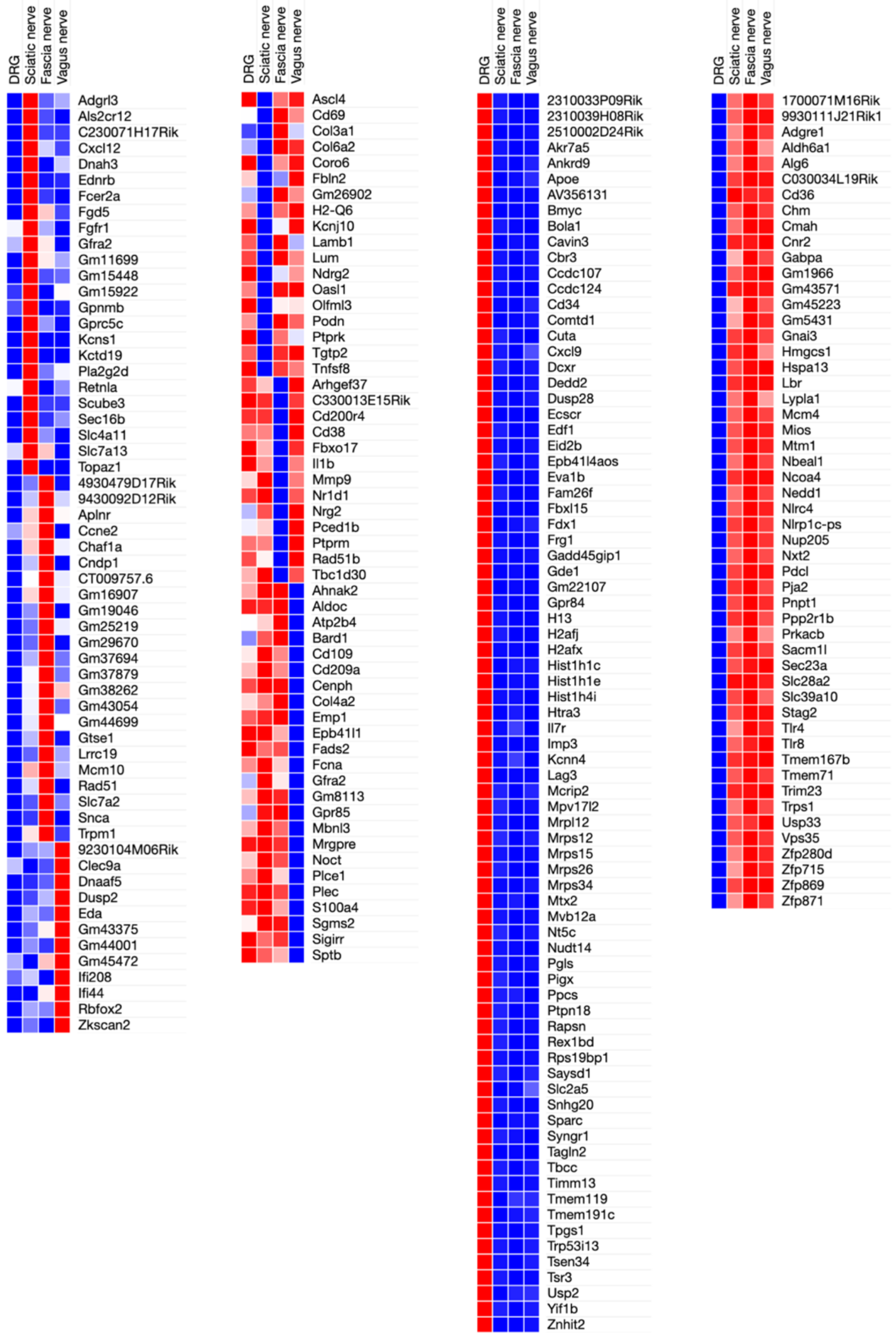


**Supplementary Figure 8. Unique gene expression patterns in individual PNS macrophage populations.** Heat map of upregulated and downregulated mRNA transcripts in single PNS macrophage populations by fourfold or more in sciatic, fascial, and vagal nerves and eightfold or more in DRG relative to their expression in the remaining three populations combined. Each box represents average of three replicates.

**Supplementary Figure 9. Expression of microglial homeostatic genes in brain and spinal cord microglia**

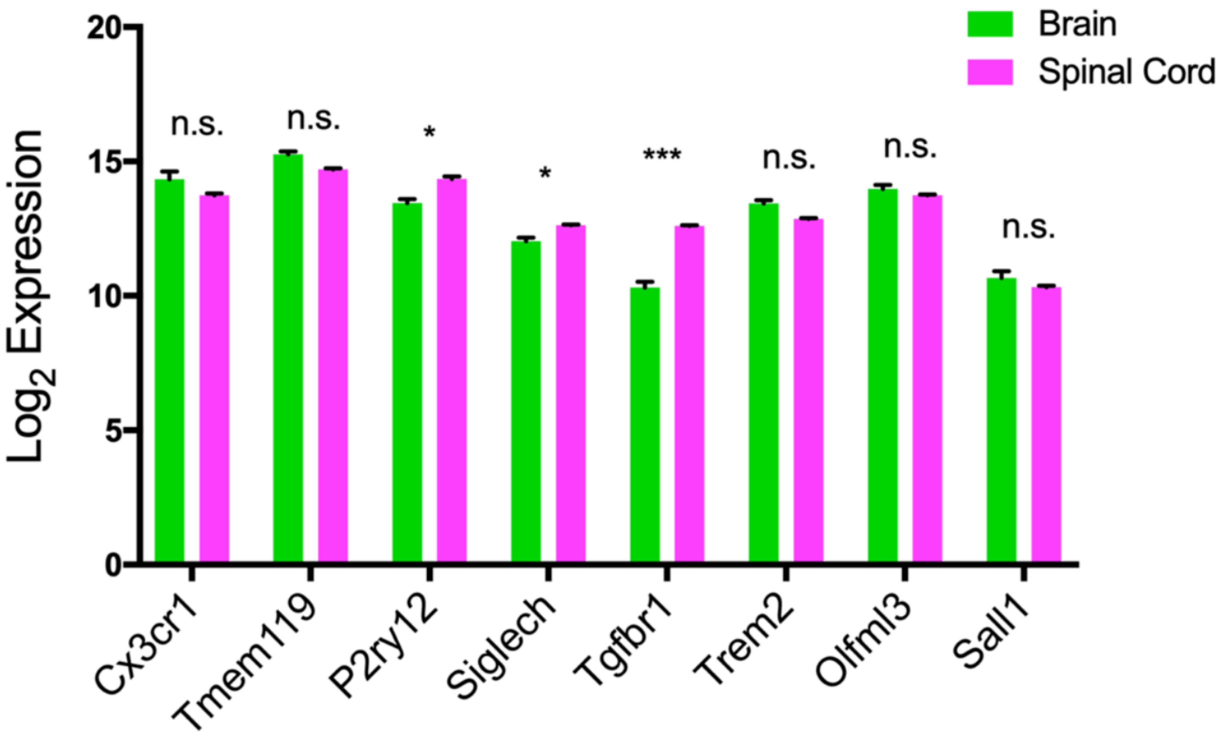


**Supplementary Figure 9. Expression of microglial homeostatic genes in spinal cord and brain microglia**. $Log_2$ expression of microglial homeostatic genes in brain (green) and spinal cord (magenta) microglia. Multiple t tests. Data are mean of three replicates +/- SEM with adjusted p-value shown. * $P < 0.05$, ** $P < 0.01$, *** $P < 0.001$.

**Supplementary Figure 10. Flow cytometric gating of blood and CNS populations in Flt3-Cre LSL-YFP**

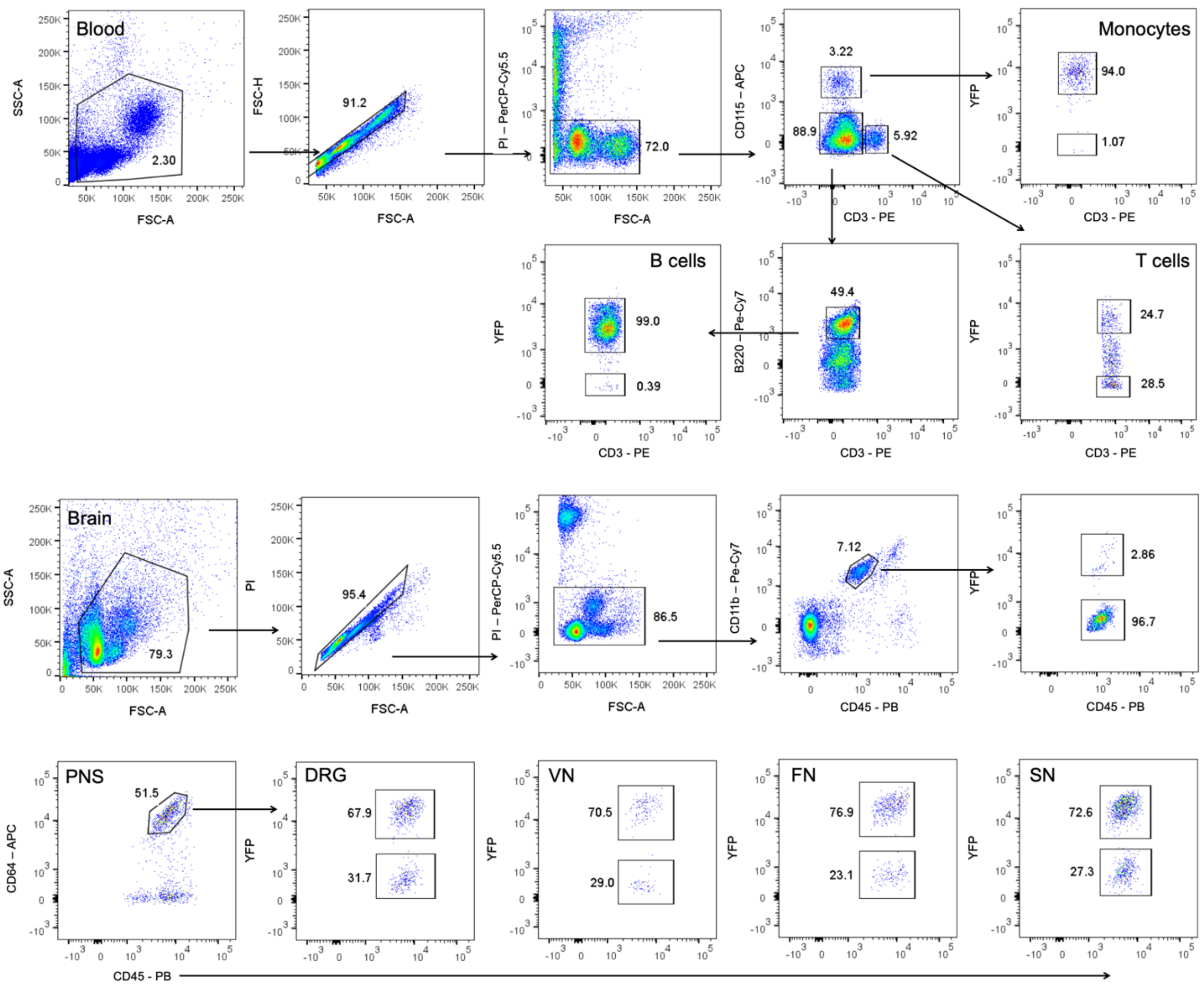


**Supplementary Figure 10. Flow cytometric gating of blood and CNS populations in Flt3-Cre LSL-YFP**. Gating strategy for determining YFP- and YFP+ percentages in monocytes, B cells, T cells, brain microglia, and PNS macrophages (related to Figure 5a-b).

**Supplementary Figure 11. Embryonic day 8.5 labeling in blood and non-neuronal tissues**

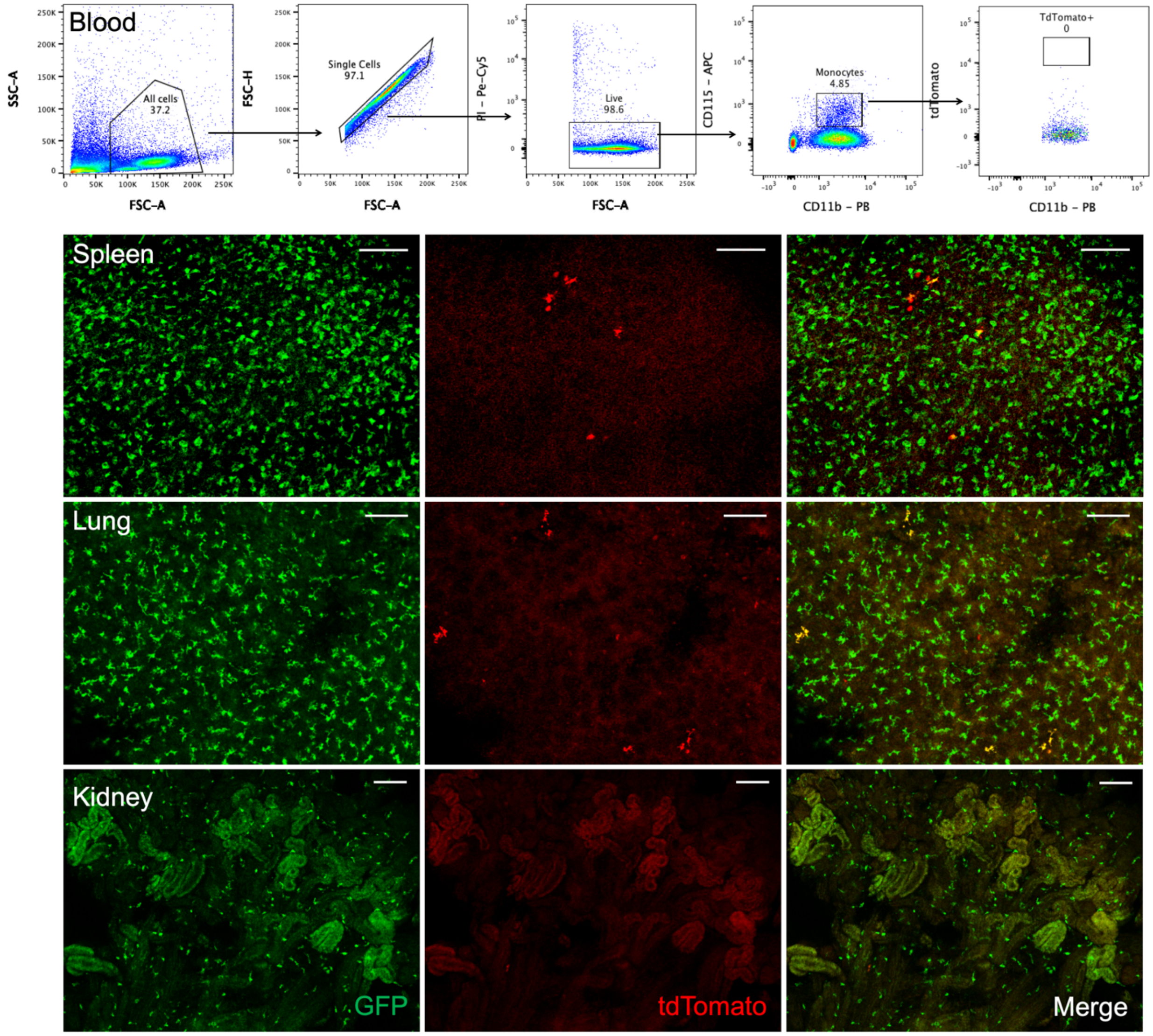


**Supplementary Figure 11. Embryonic day 8.5 labeling in blood and non-neuronal tissues**. Flow cytometric gating of tdTomato labeling in blood and representative imaging of spleen, lung, and kidney of CSF1R$^{Mer\text{-}iCre\text{-}Mer}$ x tdTomato$^{fl/fl}$ x CX3CR1-$^{GFP/+}$ newborn pups pulsed with tamoxifen at embryonic day 8.5. Scale bar, 100 μm.

**Supplementary Figure 12. CCR2 is not required for seeding and maintaining PNS macrophages (related to Figure 5f, g)**

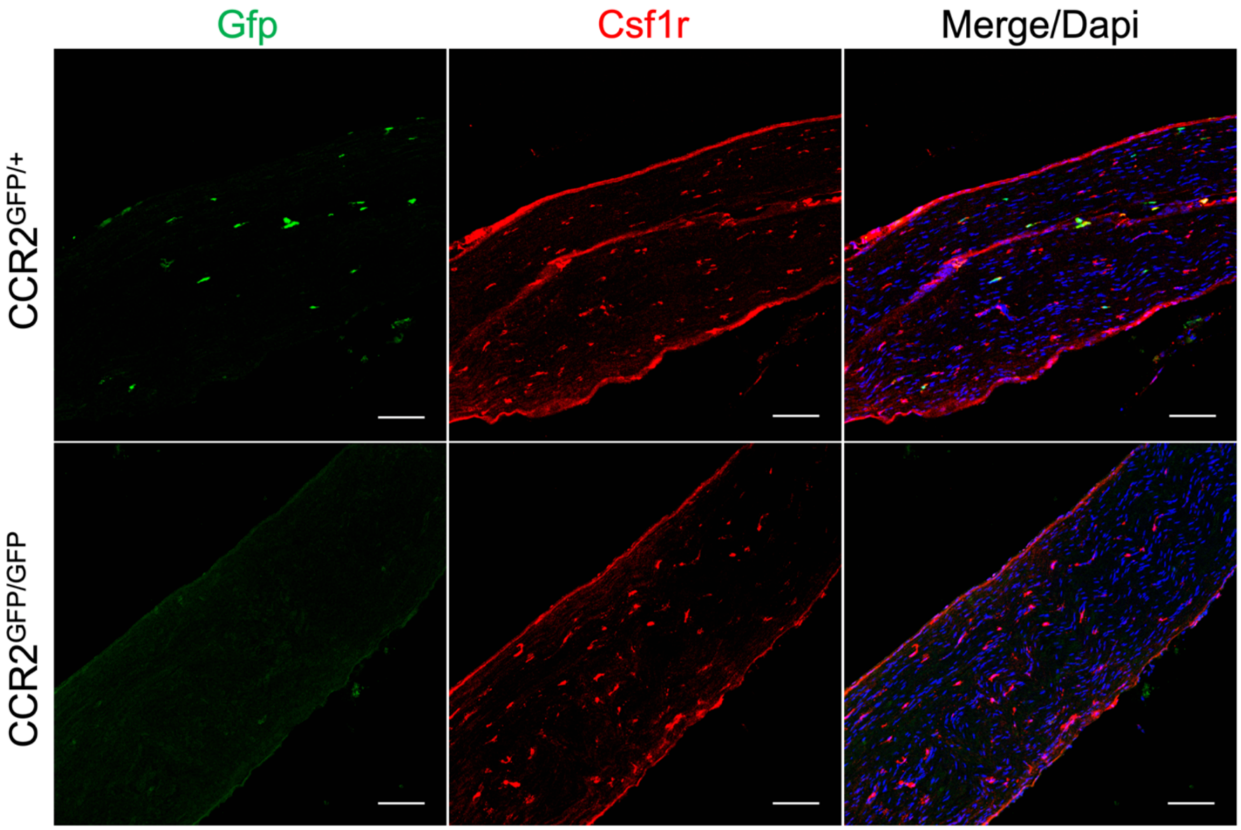


**Supplementary Figure 12. CCR2 is not required for seeding and maintaining PNS macrophages (related to Figure 5f, g)**. Representative imaging of total CSF1R+ and CCR2+ macrophages in sciatic nerve sections of $CCR2^{GFP/+}$ and $CCR2^{GFP/GFP}$ mice. Scale bar, 100 μm.

**Supplementary Figure 13. DRG macrophages are reduced in IL-34 KO mice (related to Figure 5j, k)**

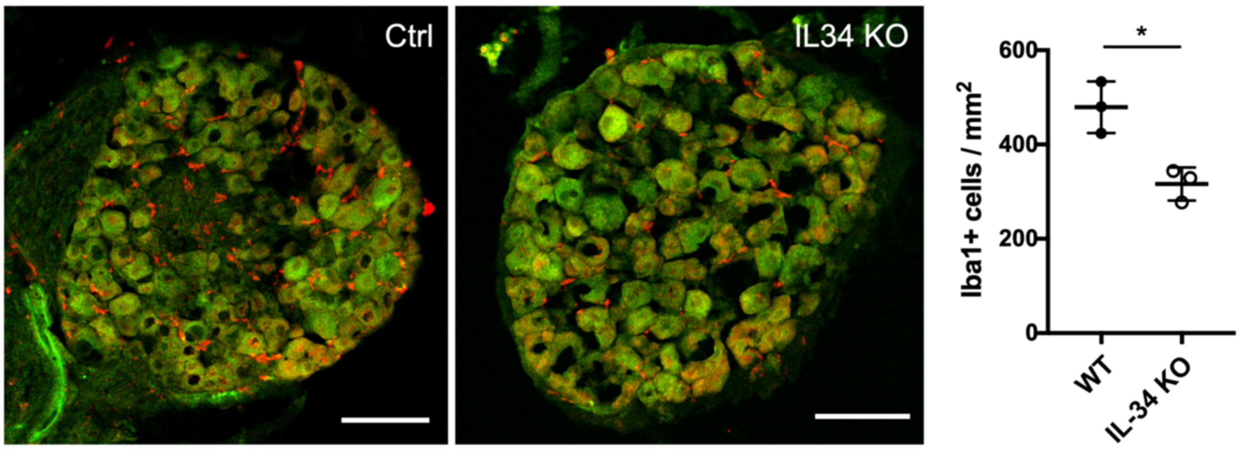


**Supplementary Figure 13. DRG macrophages are reduced in IL-34 KO mice (related to Figure 5j, k)**. Representative imaging of Iba1+ macrophages (red) in DRG and quantification of macrophages. At least 3 images were analyzed and averaged for each DRG (n = 3 mice per group). Scale bar, 100 µm. Unpaired t test. Data are mean +/- SD.

**Supplementary Figure 14. Quantification of CCR2+ subsets in YFP+ and YFP- macrophages in Flt3-Cre LSL-YFP mice (related to Figure 6f)**

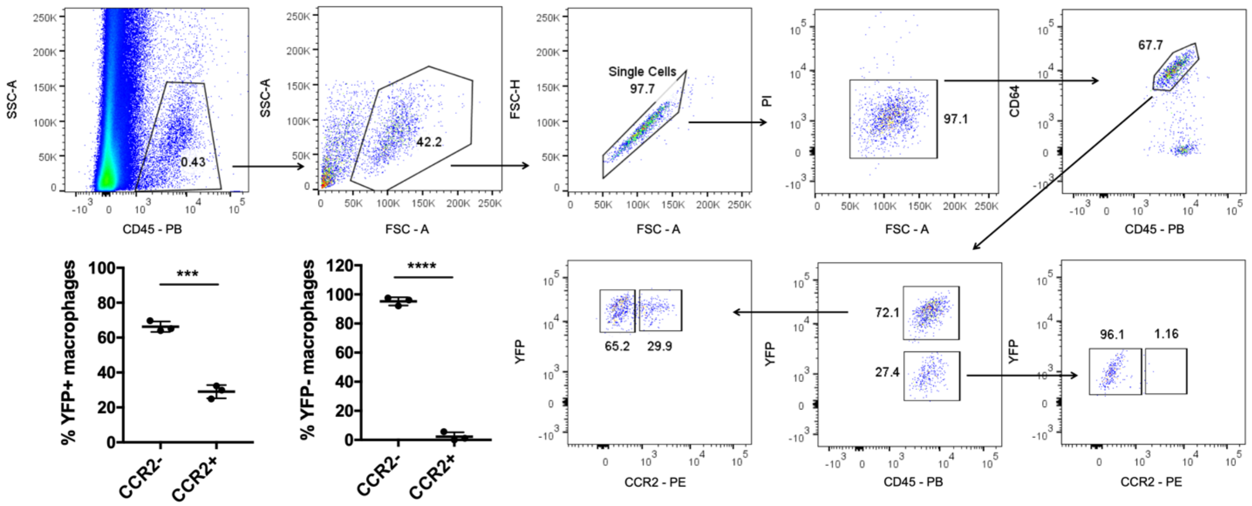


**Supplementary Figure 14. Quantification of CCR2+ subsets in YFP+ and YFP- macrophages in Flt3-Cre LSL-YFP mice (related to Figure 6f)**. Gating scheme for identification and analysis of CCR2+ and CCR2- macrophages as a percentage of both YFP+ and YFP- macrophages in Flt3-Cre LSL-YFP mice. Data are mean +/- SD (n = 3).
