# Supplementary material for: Peripheral nerve resident macrophages share tissue-specific programming and features of activated microglia
Source: Nat Commun. 2020 May 21;11:2552. doi: 10.1038/s41467-020-16355-w (PMC7242366; doi:10.1038/s41467-020-16355-w)
Supplement: Supplementary file 3 — Description of Additional Supplementary Files [file 41467_2020_16355_MOESM3_ESM.pdf]

Description of Additional Supplementary Files

Supplementary Data 1: RNA-seq analysis of PNS, CNS, and conventional macrophage populations. Tab 1 depicts expression values for all genes assessed. Tab 2 records statistically significant, differentially expressed genes.

Supplementary Data 2: Gene ontology analysis of shared and unique genes in PNS macrophages (related to Figure 2d, e)

Supplementary Data 3: Enriched genes from CNS microglia and PNS macrophages (related to Figure 3a)

Supplementary Data 4: Disease-associated gene connectivity in PNS macrophages and CNS microglia (related to Figure 3b, c)

Supplementary Data 5: Cluster-specific genes from single cell sequencing (related to Figure 6)
