# Supplementary material for: Peripheral nerve resident macrophages share tissue-specific programming and features of activated microglia
Source: Nat Commun. 2020 May 21;11:2552. doi: 10.1038/s41467-020-16355-w (PMC7242366; doi:10.1038/s41467-020-16355-w)
Supplement: Supplementary file 9 — Reporting Summary [file 41467_2020_16355_MOESM9_ESM.pdf]

nature research

Corresponding author(s): Gwendalyn J. Randolph

Last updated by author(s): Mar 28, 2020

Reporting Summary

Nature Research wishes to improve the reproducibility of the work that we publish. This form provides structure for consistency and transparency in reporting. For further information on Nature Research policies, see Authors & Referees and the Editorial Policy Checklist.

Statistics

For all statistical analyses, confirm that the following items are present in the figure legend, table legend, main text, or Methods section.

n/a	Confirmed	
☐	☒	The exact sample size (n) for each experimental group/condition, given as a discrete number and unit of measurement
☐	☒	A statement on whether measurements were taken from distinct samples or whether the same sample was measured repeatedly
☐	☒	The statistical test(s) used AND whether they are one- or two-sided<br>*Only common tests should be described solely by name; describe more complex techniques in the Methods section.*
☐	☒	A description of all covariates tested
☐	☒	A description of any assumptions or corrections, such as tests of normality and adjustment for multiple comparisons
☐	☒	A full description of the statistical parameters including central tendency (e.g. means) or other basic estimates (e.g. regression coefficient) AND variation (e.g. standard deviation) or associated estimates of uncertainty (e.g. confidence intervals)
☐	☒	For null hypothesis testing, the test statistic (e.g. F, t, r) with confidence intervals, effect sizes, degrees of freedom and P value noted<br>*Give P values as exact values whenever suitable.*
☒	☐	For Bayesian analysis, information on the choice of priors and Markov chain Monte Carlo settings
☐	☒	For hierarchical and complex designs, identification of the appropriate level for tests and full reporting of outcomes
☒	☐	Estimates of effect sizes (e.g. Cohen's d, Pearson's r), indicating how they were calculated

Our web collection on statistics for biologists contains articles on many of the points above.

Software and code

Policy information about availability of computer code

Data collection	For bulk RNA-seq, library preparation, RNA-sequencing, data generation and quality-control was conducted by ImmGen according to the consortium’s standard protocols (https://www.immgen.org/Protocols/ImmGenULI_RNAseq_methods.pdf).A total of 4,500 Flt3-negative and 16,000 Flt3-positive cells were loaded to separate lanes of the 10X Chip for preparation of two single-cell libraries. For single cell RNA-seq, the library preparation was performed according to the manufacturer’s instructions (Chromium Single-cell v2; 10X Genomics, USA).
Data analysis	For bulk RNA-seq, the reads were aligned to the mouse genome GRCm38/mm10 primary assembly and gene annotation vM16 using STAR 2.5.4a. The raw counts were generated by using featureCounts (http://subread.sourceforge.net/). Normalization was performed using the DESeq2 package from Bioconductor. Differential gene expression analysis was performed using edgeR 3.20.9 in a pairwise manner among all conditions, and a total of 12,241 differentially expressed genes were defined with a p-value <= 0.001 and > 4-fold difference. To construct the correlation plot, Euclidean distance among samples were calculated based on the differential expression matrix and clustering was performed using the ward.D2 algorithm in R.<br><br>For single cell RNA-seq, reads were mapped by using the cellranger pipeline v2.1.1 onto the reference genome grcm38/mm10. We filtered cells for those with ≥50,000 mapped reads, leaving ~1k Flt3 negative and 4k Flt3 positive cells. Downstream analyses were performed by using the package Seurat2 and Monocle in R.

For manuscripts utilizing custom algorithms or software that are central to the research but not yet described in published literature, software must be made available to editors/reviewers. We strongly encourage code deposition in a community repository (e.g. GitHub). See the Nature Research guidelines for submitting code & software for further information.

Data

Policy information about availability of data

All manuscripts must include a data availability statement. This statement should provide the following information, where applicable:

- Accession codes, unique identifiers, or web links for publicly available datasets
- A list of figures that have associated raw data
- A description of any restrictions on data availability

Sequencing reads and expression data are submitted in NCBI GEO database

Field-specific reporting

Please select the one below that is the best fit for your research. If you are not sure, read the appropriate sections before making your selection.

☒ Life sciences ☐ Behavioural & social sciences ☐ Ecological, evolutionary & environmental sciences

For a reference copy of the document with all sections, see nature.com/documents/nr-reporting-summary-flat.pdf

Life sciences study design

All studies must disclose on these points even when the disclosure is negative.

Sample size	No sample size calculation was performed, bulk RNA-seq were performed according to ImmGen protocol.
Data exclusions	No data were excluded from analyses.
Replication	Triplicates were performed for each bulk RNA-seq conditions according to ImmGen standard protocol.
Randomization	Experimental groups were determined by mouse genotype.
Blinding	No blinding was used for experiments or analyses.

Reporting for specific materials, systems and methods

We require information from authors about some types of materials, experimental systems and methods used in many studies. Here, indicate whether each material, system or method listed is relevant to your study. If you are not sure if a list item applies to your research, read the appropriate section before selecting a response.

Materials & experimental systems

n/a	Involved in the study	
☐	☒	Antibodies
☒	☐	Eukaryotic cell lines
☒	☐	Palaeontology
☐	☒	Animals and other organisms
☒	☐	Human research participants
☒	☐	Clinical data

Methods

n/a	Involved in the study	
☒	☐	ChIP-seq
☐	☒	Flow cytometry
☒	☐	MRI-based neuroimaging

Antibodies

Antibodies used	B220 (clone RA3-6B2), CCR2 (clone SA203G11), CD3e (clone 145-2C11), CD4 (clone RM4-5), CD8 (clone 53-6.7), CD11b (clone M1/70), CD16 (clone 2.4G2), CD45 (clone 30-F11), CD64 (clone X54-5/7.1), CD115 (clone AFS98), GR1 (clone 1A8), Ly-6C (clone HK1.4), MHCII (IA/IE clone M5/114.15.2), CSF1R (R&D Systems, Accession # P09581), LYVE1 (ab14917), and Clec7a (clone R1-8g7)
Validation	All antibodies used were previously validated by the manufacturer and/or in publications.

Animals and other organisms

Policy information about studies involving animals; ARRIVE guidelines recommended for reporting animal research

Laboratory animals	The following mouse strains were used: C57/B6, CX3CR1GFP/+, CX3CR1-CreER Rosa26tdTomato, CX3CR1-GFP/+ MPZ-Cre Rosa26TdTomato, Flt3-Cre LSL-YFP, IL34LacZ/LacZ, and Ccr2GFP/GFP. For Immgen sorts, 6 week-old male C57/B6 mice were used. For imaging and flow cytometric characterization of PNS macrophages, 6-12 weeks-old CX3CR1GFP/+ mice were used. For

pulse chase experiments, male and female CX3CR1-CreER Rosa26tdTomato mice 8-48 weeks-old mice were used. For lineage tracing and single cell analysis, 8-16 weeks old male Flt3-Cre LSL-YFP mice were used. For IL34-/- and CCR2-/- experiments, 10-12 week old male mice were used.

Wild animals	N/A
Field-collected samples	N/A
Ethics oversight	Mouse care and experiments were performed in accordance with protocols approved by the Institutional Animal Care and Use Committee (IACUC) at Washington University in St. Louis under the protocols 20170154 and 20170030.

Note that full information on the approval of the study protocol must also be provided in the manuscript.

Flow Cytometry

Plots

Confirm that:

☒ The axis labels state the marker and fluorochrome used (e.g. CD4-FITC).

☒ The axis scales are clearly visible. Include numbers along axes only for bottom left plot of group (a 'group' is an analysis of identical markers).

☒ All plots are contour plots with outliers or pseudocolor plots.

☒ A numerical value for number of cells or percentage (with statistics) is provided.

Methodology

Sample preparation	For nerves and all other tissues, mice were sacrificed and perfused with PBS. Nerves were harvested and kept on ice until dissociation. For ImmGen samples, nerves from 4-20 mice were pooled for each replicate. Cells were then incubated with gentle shaking for 20 minutes in digestion media containing collagenase IV, hyaluronidase, and DNAse. Cells were then washed and filtered through 70uM cell strainers. For brain and spinal cord, myelin was removed using a 40/80% Percoll gradient. Single-cell suspensions were stained at 4C.
Instrument	FACSAria II (sorting) and LSRII (analysis) by Becton Dickinson
Software	FlowJo
Cell population abundance	To ensure purity for bulk RNA-seq, cells were double sorted for a final count of 1000 cells into lysis buffer according to ImmGen protocol.
Gating strategy	Populations were first defined by size and side scatter using FSC/SSC gating. Next, singlets were determined and dead cells were excluded using PI staining. Finally, PNS macrophages were determined by gating on CD45 (intermediate) and Cx3cr1-GFP+ or CD64+ cells.

☒ Tick this box to confirm that a figure exemplifying the gating strategy is provided in the Supplementary Information.
